# Knowledge of cardiovascular disease risk and exercise duration among asymptomatic sedentary male individuals participating in Islamic prayer (Salaah)

**DOI:** 10.1186/s13102-022-00449-7

**Published:** 2022-04-01

**Authors:** Abdul Hamid Jalal, Habib Noorbhai

**Affiliations:** Department of Sport and Movement Studies, Faculty of Health Sciences, University of Johannesburg, Office 6400H, 6th Floor, John Orr Building, Doornfontein Campus, Doornfontein, Johannesburg, South Africa

**Keywords:** Salaah, Cardiovascular disease, Risk factors, Sedentary behaviour, Exercise

## Abstract

**Background:**

This study aimed to investigate the knowledge of cardiovascular disease risk among asymptomatic sedentary males participating in Islamic prayer, alongside various exercise durations and age groups.

**Methods:**

A cross-sectional study design was used. Sedentary male participants (n = 243) completed an online 78-point self-administered CVD risk questionnaire. Descriptive and inferential statistical methods were used to determine the research findings. For statistical rigour, participants were divided into two age groups. Participants were divided into three categories based on current exercise durations. Inter-group comparisons were completed using a one-way ANOVA, Kruskal–Wallis and Mann–Whitney test. The Pearson correlation coefficient was used to explore significant relationships. All statistical analyses were conducted using SPSS (Version 26, IBM). The level of significance was set at *p* < 0.05.

**Results:**

The 21–30 age group 71.09% (7.53) and the 31–40 age group 72.74% (5.53) presented with Knowledge of CVD risk and prevention scores which indicated that older individuals were more knowledgeable about CVD risk and prevention. A significant difference [95% CI (− 6.76: 1.28), (*p* = 0.002)] existed among the 10–60-min and 61–140-min exercise duration categories. A significant difference (*p* = 0.006) was also found for inter-group comparisons. This result confirmed variability with duration categories. Significant differences were found between overall knowledge of CVD scores within the two age groups (*p* = 0.03). A negative correlation was demonstrated regarding knowledge of CVD risk and prevention, and duration of exercise (r = − 0.252; *p* = 0.000).

**Conclusions:**

Knowledge about CVD risk and prevention is crucial for understanding the risk factors for CVD. The older individuals become, the more knowledgeable they are of CVD risk and prevention factors. Results show more inactive people are less likely to seek out knowledge of CVD risk and prevention. The study recommends that sedentary populations should engage in public health information interventions, physical activity (such as Salaah) and healthy lifestyle modifications. This will inform, increase awareness, and improve understanding of prevention strategies and risk factors for CVD.

## Background

Global healthcare systems are greatly burdened by cardiovascular disease (CVD) risks particularly in public health settings [1]. The CVD risk factors (due to the lack of knowledge and understanding thereof) has created a double burden for many countries worldwide. Subsequent research has taken interests in investigating varied ethnic sedentary groups of people among populations globally [2]. Of particular interest, is the under-studied sedentary Muslim male group who perform the daily Islamic prayer (Salaah) as a form of physical activity.

‘Salaah’ is the Arabic term for the Islamic obligatory prayers. These are performed five times daily by members of the Islamic faith [3]. Each prayer is made up of bouts of repetitive movements and postures along with transitions. The Salaah is accompanied by recitations of the Islamic religious text (the Quraan). These prayers are performed in units, or sets termed as a Rakaat in Arabic. A single Rakaat follows a strict sequence of movements. It begins with the standing upright posture accompanied by a recitation. At the end of the recitation, the individual bows at the hips flexing their trunk forward. Thereafter, moving into the upright position, the individual kneels onto their knees to prostrate. This is done by placing their palms and forehead flat on the ground with their elbows bent. The final posture in the sequence includes the sitting posture. The individual sits down on their flexed knees and keeps their trunk upright. To conclude the single Rakaat, the individual will then transition into the next sequence by moving into the standing posture again [4].

Global diversity of populations, including South Africa exist along with various types of exercise. The exercises are unique to certain religions and cultures, i.e., Yoga, Tai-chi and even Salaah[5]. These are also well researched as moderate-to-low intensity aerobic exercise types. The Salaah, like most moderate-to-low intensity exercise types includes static postures and flexibility movements. Functional strength transitions are included with the postures that can be scientifically measured, similarly to other exercise modalities [6].

Previous studies recommend that regular moderate-to-low intensity physical activity is beneficial to abating CVD [7]. Knowledge, awareness and understanding of CVD are fundamental in improving contemporary status of the health of sedentary populations [8]. This study aimed to fill an information void by documenting the knowledge levels of CVD risk and prevention in sedentary male individuals, who perform the Islamic prayer daily.

### Objectives

Risk screening and measurement are important components in managing public healthcare systems [9]. Some measurement tools include self-reported surveys or risk screening questionnaires [10]. It is, therefore, crucial that risk screening be conducted on as many diverse sedentary groups within any population. To address the specific gap in CVD research for individuals who perform the Salaah, the rationale of the study was to explore this cohorts’ current levels of knowledge, understanding and awareness of CVD.

## Methods

### Study design

This study employed a cross-sectional design using convenience sampling and volunteer participants. The study used an anonymous 78-point CVD risk questionnaire to fulfil the research objective and quantify the current levels of knowledge and understanding of CVD in this sedentary population.

### Study participants

A total of 243 anonymous male participants aged 21–40 years volunteered for this study. These participants were divided into experimental Group 1 (21–30-year-olds) and Group 2 (31–40-year-olds) for the purposes of statistical analysis. Responses were gathered from participants in South Africa. To be included into the study, the criteria included only asymptomatic sedentary Muslim males who had no current chronic or orthopaedic pathologies. All participants performed less than 150 min of total exercise per week. Participants of the study did not exercise for a minimum of four months or entirely before answering the questionnaire. The participants were also asked to verify their activity level upon recruitment. Only males were chosen for this study. Females have different genetics, and their postures differ in comparison to males in the Salaah according to the teachings of the Islamic faith. Male volunteer participants were also more willing to engage with the researcher on this topic during recruitment.

### Data collection

The self-administered, online CVD risk questionnaire chosen was validated for public health screening purposes by Woringer et al. [11] in the United Kingdom. It was chosen since it provided valuable knowledge-based questions about the understanding of CVD risk for groups of sedentary individuals. The n = 243 volunteer participants completed the online questionnaire by means of an electronic email link using GoogleForms™. The link was communicated through social media platforms on WhatsApp™ and Instagram™. The advantage of using the GoogleForms™ tool was that responses were automatically captured on a GoogleSpreadsheet™ and was access-controlled by the researcher. Each participant agreed to participate through an informed consent section provided on the questionnaire. There was no physical study setting applicable to this type of research study.

The questionnaire comprised of nine sections. These questions assessed the current CVD knowledge of participants, their awareness of cardiovascular disease, and certain practices of CVD risk factors. The first section gathered participants’ demographics and their existing activity status (total minutes of exercise they complete per week). Scores were allocated to correct answers that measured the current knowledge and awareness of CVD risk and prevention. The eight sections of the questionnaire that followed consisted of a Likert scale response selection with closed-ended questions. Participants allocated answers to questions by choosing various options, namely: 1—strongly disagree, 2—disagree, 3—agree and 4—strongly agree. These choices were available for all sections except for section one and seven of the questionnaire. The response choices for section seven were 1—not at all confident, 2—somewhat confident, 3—moderately confident, 4—very confident and 5—completely confident [11]. All items relating to alcohol consumption from the original 85 point validated questionnaire were removed. These questions were not applicable to the Muslim population who are prohibited from consuming alcohol according to the Islamic faith.

### Data analysis

The statistical analysis software package for social sciences (SPSS), Version 26 (IBM, United States), was used for the analysis of the study results [12]. The study made use of descriptive and inferential statistical analyses. Study population demographics were described using descriptive statistics. Means and standard deviations were measured through data variance. Descriptive analysis was also used to analyse data between age groups and exercise categories. For added riguor in the descriptive analysis, the 243 participants were divided into three categories according to their current exercise participation duration (0–9 min, 10–60 min, and 61–140 min). Participants were also compared in terms of the two age groups as mentioned previously (21–30 years and 31–40 years). Inter-group comparisons were computed using a one-way ANOVA (analysis of variance), Kruskal–Wallis test and a Mann–Whitney test. Inferential statistical analysis using the Pearson’s correlation coefficient was computed to explore relationships between the levels of knowledge of CVD risk and prevention with other variables of the study.

Since the total sample of participants were more than fifty, the study used the Kolmogorov–Smirnov test for normality. Data was taken from a normal distribution of the population. Only parametric testing was considered since no outliers were presented that would distort the mean and standard deviations. The Pearson correlation coefficient was used to determine any statistically significant relationships between the variables of the survey. The level of significance was set at *p* < 0.05.

### Ethical considerations

All methods were carried out in accordance with relevant guidelines and regulations. An online information sheet explaining the research and a consent form was attached to the questionnaire. This sheet was obtained from all participants prior to the commencement of the data collection. Since the questionnaire had been used in previous literature, there was no potential harm that participants would face by completing it. Participants provided consent before participating. All responses were completely anonymous and privacy was maintained. Ethical approval for this study was granted by the Faculty of Health Sciences Research Ethics Committee (REC-474-2020) at the University of Johannesburg, South Africa.

## Results

### Participant demographics

The results of the study presented 128 participants (52.70%), aged 21–30 years in group 1, whilst 115 participants (47.30%) aged 31–40 years were found in group 2 (Table 1). The variables of the study included knowledge of CVD risk and prevention, perceived benefits, perceived barriers, self-efficacy, and intentions to change behaviour or cues to action. Reported scores of the knowledge of CVD risk and prevention data were collected from both groups of participants. Group 1 presented with a reported knowledge of CVD risk and prevention score of 71.09% (7.53) whereas group 2 presented with a score of 72.74% (5.53).

**Table 1:** Age demographics of participants

Age groups	Frequency	Percent (%)
21–30 years	128	52.7
31–40 years	115	47.3
Total	243	100

### Exercise minutes performed in a week

**Fig. 1:**
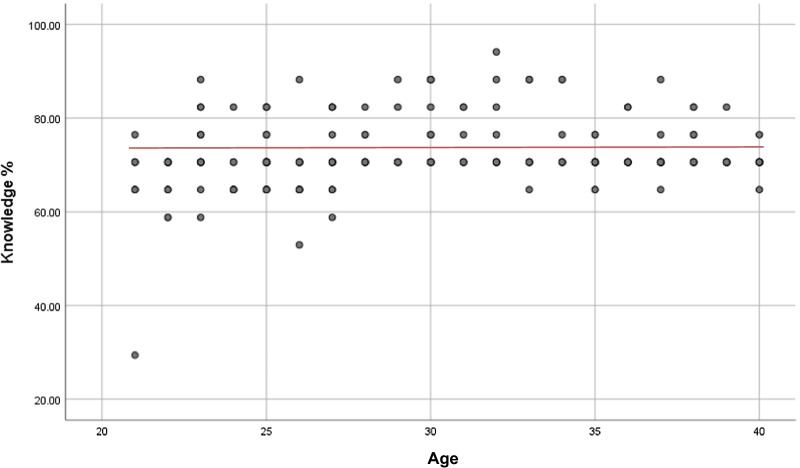
Scatter plot displaying the overall reported knowledge of CVD risk and prevention scores amongst the different ages of participants

The overall reported knowledge of CVD risk and prevention scores of the study was 71.87% (± 6.69). In terms of age, group 2 reported greater perceived knowledge of CVD risk 72.74% (± 5.53) compared to group 1 71.09% (± 7.53). The maximum score among all participants was 94.12% and the minimum score being 29.41%, respectively. Figure 1 displays the overall reported knowledge of CVD risk and prevention scores amongst the participants.

Based on exercise duration, no participants completed 150 min or more of exercise as this would have classified them as non-sedentary [13]. A categorisation was made for participants who exercised for 0–9 min (11.1%), 10–60 min (24.3%) and 61–140 min (64.6%). The 10–60 min (88.9%) category who held the most amounts of participants in the study. It was found that majority of the participants exercised for 120-min (25.5%).

### Inter-group comparisons

For inter-group comparisons, a one-way analysis of variance (ANOVA) test was used to analyse the difference between the three exercise duration categories. The purpose of the one-way ANOVA was to compare if the means of the samples were statistically significant for the total knowledge of CVD risk and prevention scores. Among the 10–60-min category and the 61–140-min category, the results were statistically significant [95% CI (− 6.76: 1.28), (*p* = 0.002)].

In addition, the Kruskal–Wallis test, was used to determine any statistically significant differences of the knowledge scores between the three exercise duration categories. The results were also found to be statistically significant (*p* = 0.006), confirming that at least one sample between the three exercise duration categories stochastically dominated the other two categories in terms of inter-group variability. The Mann–Whitney test was used to determine statistically significant differences of the age groups (*p* = 0.03). This aided in comparing the overall knowledge of CVD scores among the 21–30-year-old and 31–40-year-old participants. A statistically significant difference was found for the 10–60-min and 61–140-min exercise duration categories (*p* = 0.001). Collectively, these results showed participants in the 61–140-min exercise group performed better in terms of the knowledge of CVD compared to the other two exercise categories.

### Correlations

**Fig. 2:**
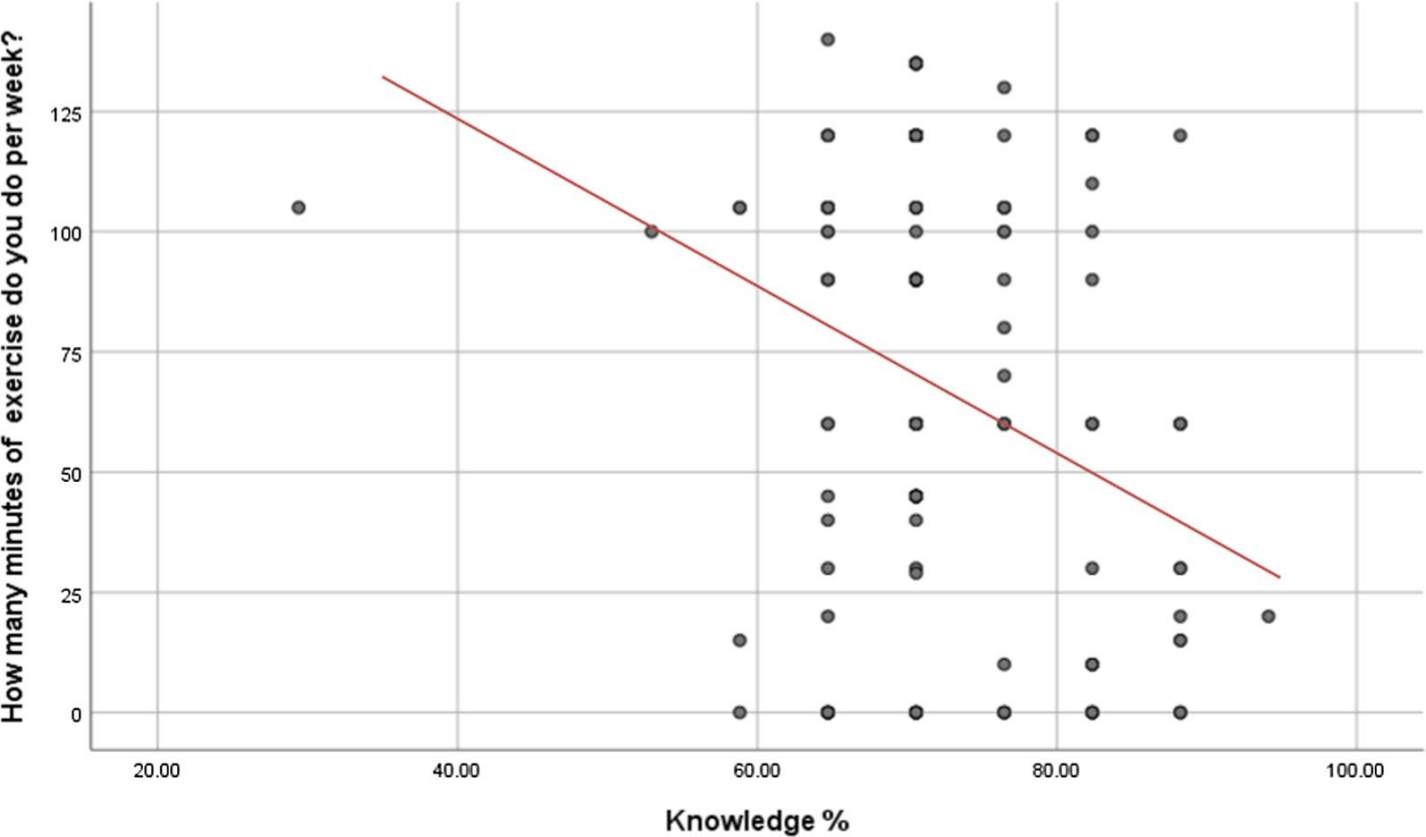
Scatter plot displaying the relationship of exercise duration in minutes per week and knowledge of CVD risk and prevention

Using the Pearson’s correlation, several relationships were investigated. A negative correlation was demonstrated for knowledge of CVD risk and prevention and duration of exercise (r = − 0.252; *p* = 0.000). For exercise category 3 (70.55 ± 5.35), the data was less positively skewed (0.19; standard error: 0.43) when compared to category 1 (r = 1.64) and category 2 (r = 1.04) (Fig. 2).

**Fig. 3:**
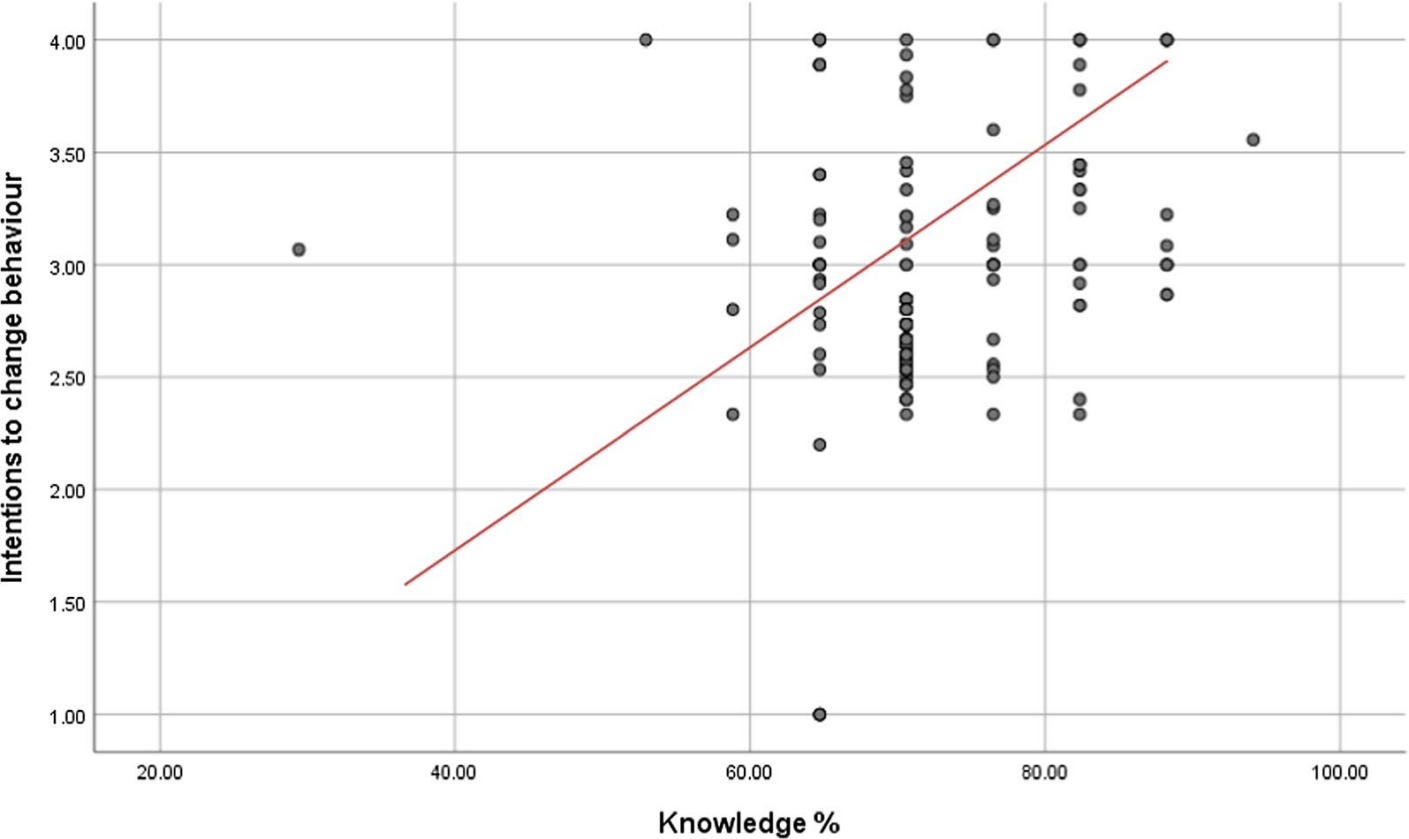
Scatter plot displaying the intentions to change behaviour and knowledge of CVD risk and prevention

Intentions to change behaviour (r = 0.255; *p* = 0.00) and perceived benefits (r = 0.239; *p* = 0.00) were positively correlated along with knowledge of CVD risk and prevention (Fig. 3). This correlation was statistically significant. Self-efficacy and perceived barriers (r = − 0.108; *p* = 0.092) displayed an inverse relationship which was not statistically significant. An inverse and significant correlation was found for perceived susceptibility (r = 0.164; *p* = 0.011) and perceived barriers (r = − 0.163; *p* = 0.011).

## Discussion

The aim of this research was to assess the levels of knowledge, understanding and awareness of CVD risk and prevention amongst sedentary, asymptomatic male individuals who perform the Salaah daily. A primary finding of this study was the evidence of a relationship between knowledge and participation in exercise. The second primary finding included the positive relationship for age and knowledge of CVD through the lifespan. Secondary research findings included the correlations of knowledge of CVD risk with perceived benefits, intentions to change behavior. The inverse relationship of knowledge of CVD with perceived barriers was also a significant research finding. Another secondary finding was the inverse relationship found among perceived barriers and self- efficacy. Each variable was explored for the most significant relationships with knowledge of CVD risk and prevention.

### Knowledge of CVD risk and prevention

In terms of knowledge regarding CVD risk and prevention, it extends to the participants’ level of acumen, awareness and understanding of what leads to or places an individual at risk for cardiovascular disease [14]. The knowledge reported by participants includes nutritional information about dietary considerations that would contribute to an increased risk of cardiac pathologies. The effects of cholesterol on the blood, and healthy eating are also encompassed here. The self-reported knowledge scores for the questionnaire were also determined by other pathophysiological questions on normal blood pressure, physical activities that are considered exercise. This is in conjunction with the amount of physical activity that is beneficial for lowering an individual’s risk for CVD [15].

### Perceived benefits

Perceived benefits involve perceptions about the effectiveness that healthy lifestyle modifications may have in preventing cardiovascular disease risk. For the purposes of this study, perceptions on the effect of exercise, a healthy diet as well as smoking cessation was included. Again, these are the perceptions that the participants attached to the benefits.

### Perceived barriers

When it comes to perceived barriers, we look at physical, socioeconomic, and available resources barriers to make the appropriate lifestyle changes that counter sedentary behavior [16]. These include barriers to undertaking an exercise regimen, availability, affording healthy foods and even perceptions about what constitutes a healthy diet. These are, albeit perceived barriers that the individual has control over and are modifiable.

### Self-efficacy

The key word for the variable of self-efficacy is confidence. The survey tested each participant’s belief to implement certain healthy lifestyle modifications to attain and lower their risk profile against cardiovascular disease. According to Albert Bandura, self-efficacy is a strong measure of confidence for each individual and this allows them to control their social setting, motivation and behavior [17].

### Intentions to change behavior

The intentions to change behavior or cues to action reported scores included each participant’s willingness and desire to change their lifestyle for the better. This involves maintaining a healthy body weight, the intention to cease smoking, engaging in a healthy eating and exercise regimen and also taking the appropriate medications to combat high blood pressure and high blood cholesterol levels.

### Knowledge of CVD risk and age

Participants showed an above average overall score of perceived knowledge on cardiovascular disease risk factors. Based on the results of the study, the 31–40 year old sedentary adult males who performed the Salaah daily had reported a greater score of perceived knowledge on cardiovascular disease risk factors compared to younger individuals of the same criteria. It can be deduced, from the results that older sedentary asymptomatic male individuals are more knowledgeable about the risk of cardiovascular disease risk based on their self reported perceived knowledge score.

Previous research has shown that as people age, their risk profiles increase [18] however, this research has found that their knowledge and understanding of health risks improves. The older individuals become, the more knowledge and awareness they obtain of cardiovascular disease risk along the lifespan.

A possible research outcome of this finding would indicate that much more knowledge promotion on cardiovascular disease risk factors in public health care systems is required to be propagated to younger sedentary individuals. This finding would also open the prospect for future studies to investigate the knowledge of CVD risk among much older symptomatic participants and to assess the trajectory of these results with increasing age [19].

### Knowledge of CVD risk and duration of exercise

According to previous literature, there has always been a relationship between sedentary behavior and increased cardiovascular disease risk [20]. A negative correlation was found for exercise duration and reported knowledge of CVD. This finding indicates that the less people engaged in exercise, the less knowledge and awareness they would obtain about CVD risk and prevention factors toward their health [21].

It is, therefore, imperative that the promotion of physical activity among sedentary populations be communicated extensively. Participation in exercise, even in small amounts like the Salaah contributes to improved awareness, understanding and knowledge of CVD risk. The study and its findings also assist with elaborating on the poorly understood mechanism regarding the role of exercise participation and knowledge of CVD risk [22]. It is evident that the participants who exercised for longer durations were more knowledgeable about the effects of exercise on CVD risk and prevention.

### Knowledge of CVD risk and perceived benefits

The more knowledgeable individuals are about CVD risk, the more likely they are to be aware of the benefits of leading a healthy lifestyle. These benefits extend towards mitigating CVD risk. This is also concurrent with findings by Li et al. [23].

As a research outcome, this finding also gives a platform for future scholars to investigate possible effects of self reported knowledge of CVD risk and prevention in non-sedentary populations. This will aid in measuring the extent to which they become aware of the perceived benefits associated with exercise and maintaining a healthy lifestyle [24].

### Perceived barriers and self-efficacy

Perceived barriers have been found to be inversely correlated with self-efficacy. This implies that as the barriers to engaging in healthy lifestyle modifications and regular physical activity increase, an individual will be less motivated to engage in them. They will also have less self-confidence to do so [25]. Since these are perceptions about barriers, the relationship is still dependent on the amount of knowledge, awareness and understanding that these individuals have on CVD risk and prevention strategies.

This research finding could be further investigated based on the extent to which these barriers impede self efficacy in each sedentary individual. Self efficacy is an important tool to assess the belief in one’s ability to self-motivate and overcome barriers [26]. Informative knowledge greatly assists in improving negative perceptions about healthy lifestyle choices. The notion of ‘knowledge is power’ applies in this regard [27]. It also impacts the extent to which one may view a barrier as an obstacle that can be overcome or be seen as an opportunity.

### Knowledge of CVD risk and perceived barriers

The results further display that as the perceived barriers increase, the inverse phenomenon occurs with knowledge of CVD. This inverse relationship would imply that barriers to healthy lifestyle modifications for a sedentary individual greatly increase the likelihood that they will not be as knowledgeable about CVD [28]. This necessitates the notion that sedentary individuals need further knowledge understanding of perceived barriers to improve their current knowledge of CVD.

### Knowledge of CVD risk and intentions to change behaviour

Perceived knowledge about CVD risk and prevention has been positively correlated with intentions to change behavior or cues to action. The data is mostly skewed to the right. This implies that as people’s willingness to change their behavior increases, so too will their knowledge of CVD risk and prevention increase and improve. Preventative action in the form of physical activity and healthy lifestyle modifications are essential measures that sedentary individuals should consider when improving the status of their health [29].

This finding leads to the outcome that individuals will make a concerted effort if they are aware, understand and are knowledgeable of the benefits of regular physical activity. They would assign their perceptions of benefits based on attained knowledge, toward intentions to change behavior or cues to take preventative action in their lifestyle. Acting in the form of exercise is most favorable in abating cardiovascular and other chronic diseases [30].

### Limitations

The first limitation in this study is a respondent fatigue bias. A similar limitation has also been found in previous literature [31]. It is still unclear, to an extent that participants answered questions to the best of their ability. Notably, toward the final section of the questionnaire of intentions to change behaviour or cues to take action. This could be in part due to the length of the survey, being 78 closed ended questions in total. Secondly, the knowledge-based questions included pathophysiological terms in their line of questioning. It also unclear if most of these sedentary participants understood these pathophysiological terms when answering questions.

Although having good reliability and validity scores, another limitation of using this survey was that a larger sample could have been tested. This was recommended to gain more strength in the statistical analyses and findings. Future studies could test a larger sample and expand on these findings. Finally, the original survey did not cater for populations who did not consume alcohol. Therefore, all alcohol related questions were removed. This could have impacted the knowledge scores and all other relationships that were analysed in the study.

## Conclusion

It is evident that public health knowledge interventions about CVD risk and prevention are crucial in the analysis of relationships for any variable to understanding and becoming aware of the risk factors for CVD. The study and findings further reinforce that sedentary populations who perform the Salaah daily still need to engage in such interventions and regular physical activity. This can be enhanced alongside healthy lifestyle choices to become more informed and increase their awareness and understanding of prevention strategies and risk factors for CVD. The phenomenon of CVD risk in populations globally is constantly evolving. It is up to health care systems and public health bodies to increase their reach to multiple types of under-studied sedentary populations, and particularly to younger adults to minimise the impacts of CVD.

### Recommendations

Furthermore, future studies could be completed among asymptomatic and even symptomatic sedentary males or females who perform the Salaah as a low intensity physical activity. This will not only expand current literature about Salaah as a type of physical activity in South Africa, but it will also create awareness of the levels of understanding and the contemporary knowledge on under-represented sedentary ethnic groups globally. The study affirms the concepts and findings that public health education on sedentary behavior and CVD risk is essential. In addition, the importance of physical activity and other healthy lifestyle modifications should also become a priority, given the heightened prevalence of CVD among varied groups, globally.

## Data Availability

The datasets generated during and/or analysed during the current study are not publicly available due to regulations in the University Research Ethics Committee, but may be made available from the corresponding author on reasonable request.

## Abbreviation

CVD: Cardiovascular disease
